# Housing Characteristics and Disaster Preparedness in Low-income Populations: Age as a Moderator

**DOI:** 10.1093/geroni/igaf122.1429

**Published:** 2025-12-31

**Authors:** Zhirui Chen, Samantha Teixeira, Rebekah Coley

**Affiliations:** Boston College, Framingham, Massachusetts, United States; Boston College School of Social Work, Chestnut Hill, Massachusetts, United States; Boston College, Chestnut Hill, Massachusetts, United States

## Abstract

This study examined associations between individual- and community-level housing characteristics and disaster preparedness among low-income adults. Given the increased vulnerability of older adults to climate disasters, we also tested the moderating role of age. Data from three panels (2021-2023) of FEMA’s National Household Survey were pooled and merged with CDC’s community-level Social Vulnerability Index. The analytic sample included 6,442 low-income adults nested within 4,376 zip codes. Weighted multilevel models examined associations between housing characteristics (housing type, ownership, crowding, and cost, all assessed at both the individual and community level) and three types of disaster preparedness (perceived, actual, and financial preparedness), adjusted for individual demographics. Results showed that both individual- and community-level housing characteristics were associated with individual disaster preparedness. Specifically, renters reported lower perceived and financial preparedness relative to homeowners. Compared to individuals in single-unit homes, those in mobile home reported lower financial preparedness. Those residing in communities with higher proportions of multi-unit housing or crowding also reported lower disaster preparedness. Older adults generally exhibited better perceived and financial preparedness than younger people, but their advantages diminished when experiencing housing cost burden, residing in multi-unit apartment, or living in crowded communities. Our findings highlight the important role of housing characteristics in shaping disaster preparedness and the special needs of low-income older adults facing housing vulnerability. Social workers and community practitioners can draw on these findings to develop locally-tailored and age-responsive strategies to reduce disaster risk among low-income individuals and communities while enhancing climate and disaster resilience in an aging society.

